# A novel non-negative Bayesian stacking modeling method for Cancer survival prediction using high-dimensional omics data

**DOI:** 10.1186/s12874-024-02232-3

**Published:** 2024-05-03

**Authors:** Junjie Shen, Shuo Wang, Hao Sun, Jie Huang, Lu Bai, Xichao Wang, Yongfei Dong, Zaixiang Tang

**Affiliations:** 1 Department of Biostatistics, School of Public Health, Jiangsu Key Laboratory of Preventive and Translational Medicine for Major Chronic Non-communicable Diseases, MOE Key Laboratory of Geriatric Diseases and Immunology, Suzhou Medical College of Soochow University, Suzhou, Jiangsu 215123 People’s Republic of China; 2 Institute of Medical Biometry and Statistics, Faculty of Medicine and Medical Center-University of Freiburg, 79085 Freiburg, Germany

**Keywords:** Survival stacking, Non-negative Bayesian model, Artificial neural network

## Abstract

**Background:**

Survival prediction using high-dimensional molecular data is a hot topic in the field of genomics and precision medicine, especially for cancer studies. Considering that carcinogenesis has a pathway-based pathogenesis, developing models using such group structures is a closer mimic of disease progression and prognosis. Many approaches can be used to integrate group information; however, most of them are single-model methods, which may account for unstable prediction.

**Methods:**

We introduced a novel survival stacking method that modeled using group structure information to improve the robustness of cancer survival prediction in the context of high-dimensional omics data. With a super learner, survival stacking combines the prediction from multiple sub-models that are independently trained using the features in pre-grouped biological pathways. In addition to a non-negative linear combination of sub-models, we extended the super learner to non-negative Bayesian hierarchical generalized linear model and artificial neural network. We compared the proposed modeling strategy with the widely used survival penalized method Lasso Cox and several group penalized methods, e.g., group Lasso Cox, via simulation study and real-world data application.

**Results:**

The proposed survival stacking method showed superior and robust performance in terms of discrimination compared with single-model methods in case of high-noise simulated data and real-world data. The non-negative Bayesian stacking method can identify important biological signal pathways and genes that are associated with the prognosis of cancer.

**Conclusions:**

This study proposed a novel survival stacking strategy incorporating biological group information into the cancer prognosis models. Additionally, this study extended the super learner to non-negative Bayesian model and ANN, enriching the combination of sub-models. The proposed Bayesian stacking strategy exhibited favorable properties in the prediction and interpretation of complex survival data, which may aid in discovering cancer targets.

**Supplementary Information:**

The online version contains supplementary material available at 10.1186/s12874-024-02232-3.

## Introduction

Survival prediction using high-dimensional omics data has been a widely discussed topic in the field of precision medicine, particularly when it comes to cancer research [1–3]. Genomic data that contains abundant hereditary information largely determines the phenotype heterogeneity of cancer patients [4, 5]. In recent years, high-throughput sequence technologies facilitate the extensive application of genomic information to predict the patient’s prognosis [6]. The challenge lies in how to construct efficient and robust models for survival prediction in the context of high-dimensional data.

Regularization methods, such as Lasso, relaxed Lasso, and elastic-net, are recognized as powerful modeling tools yielding predictive and interpretable models [7]. These methods were extended to the Cox model for better handling the survival data [8]. When using genomic data, these methods construct models based on individual genes, treating them as independent predictors. However, the progression and prognosis of cancer are regulated by multiple biological signaling pathways, and thus incorporating pathway-level information into model building can be a more accurate representation of the underlying biological processes [9–11]. In this light, several extensions, such as the group Lasso (grlasso) and composite minimax concave penalty (cMCP), are able to integrate the biological pathway information as group structure into the modeling procedure [12, 13]. Besides, several attempts have been made to build pathway-based modeling strategies. Chen and Wang proposed to integrate prior defined biological pathway information and gene expression profiles for cancer prognosis [14]. Zhang et al. proposed a two-stage strategy integrating risk scores derived from pathway-based models to make cancer survival prediction [15]. Kim et al. utilized a directed random walk algorithm that navigates through the pathway network, generating an effective genomic feature extraction [16]. However, the majority of these are single-model based methods, usually leading to unstable prediction. Others employ similar concepts with the naive stacking learning.

Stacking strategy is a wise ensemble learning method that combines cross-validated (CV) predictions from multiple varied algorithms or models [17]. By leveraging the strengths of different models, stacking methods often yield more robust and accurate predictions than using a single model [18]. However, the implementation of stacking methods to survival data is more complex since the predicted survival probability is varied across time. Andrew Wey, et al. proposed using the inverse probability of censoring weighted Brier Score (IPCW-BS) as the objective function for survival stacking models based on multiple time points [19]. Golmakani and Polley assumed that candidate models were all on the condition of proportional hazards and used cross-validated negative log partial likelihood as an optimization function [20]. Robert Tibshirani, et al. demonstrated that the logistic regression estimation fitting the events of different time points is the approximate estimation of the Cox model and thus one can cast survival analysis as a stacking classification problem [21]. Ginestet, et al. proposed an ensemble procedure based on the pseudo-observation-based-AUC loss to optimally stack predictions from survival algorithms [22].

In the present study, we introduced a novel survival stacking method that integrated group structure information to improve the robustness of cancer survival prediction using high-dimensional omics data. Our approach involved grouping genomic data into multiple sub-data based on biological pathway knowledge. Sub-models were then independently trained using the features in each sub-data. In addition to a non-negative linear combination of sub-models using a traditional optimization method based on the integrated Brier Score (IBS) loss function, we also proposed a Bayesian hierarchical generalized linear model (BhGLM) using the non-negative mixture double-exponential (DE) prior, as well as an artificial neural network (ANN), to ensemble the predictions of sub-models. We compared the proposed methods to several competitors, including the widely used survival penalized method and the extensions that consider the group structures, through simulation study and real-world data application. The results showed that the proposed survival stacking strategy exhibited favorable properties in prediction and interpretability.

The paper is organized as follows: In Section 2, we presented a detailed illustration of the proposed strategy. Section 3 compared the prediction performance of the proposed method and existing methods through a simulation study. In Section 4, the proposed methods were applied to several real-world data. Lastly, Section 5 concluded the paper and discussed several critical issues related to our methods.

## Materials and methods

### Pathway-based survival stacking strategy

Supposing a right-censored survival data of *n* subjects consists of triplets {(*y*_*i*_,  *δ*_*i*_, ***x***_*i*_)}, for *i* = 1, 2, …, *n*. Denote the observed survival time *y*_*i*_ = min(*t*_*i*_, *c*_*i*_), where *t*_*i*_ and *c*_*i*_ are event time and censored time, respectively. *δ*_*i*_ = *I*(*t*_*i*_ < *c*_*i*_) indicates the occurrence of events. The goal is to estimate the survival function of the event-time random variable *Y* that depends on *p* covariates ***x***, i.e. *S*(*y*| ***x***) = *P*(*Y* > *y*| ***x***). In this study, we aim to predict the survival of cancer patients using genomics data.

**Fig. 1:**
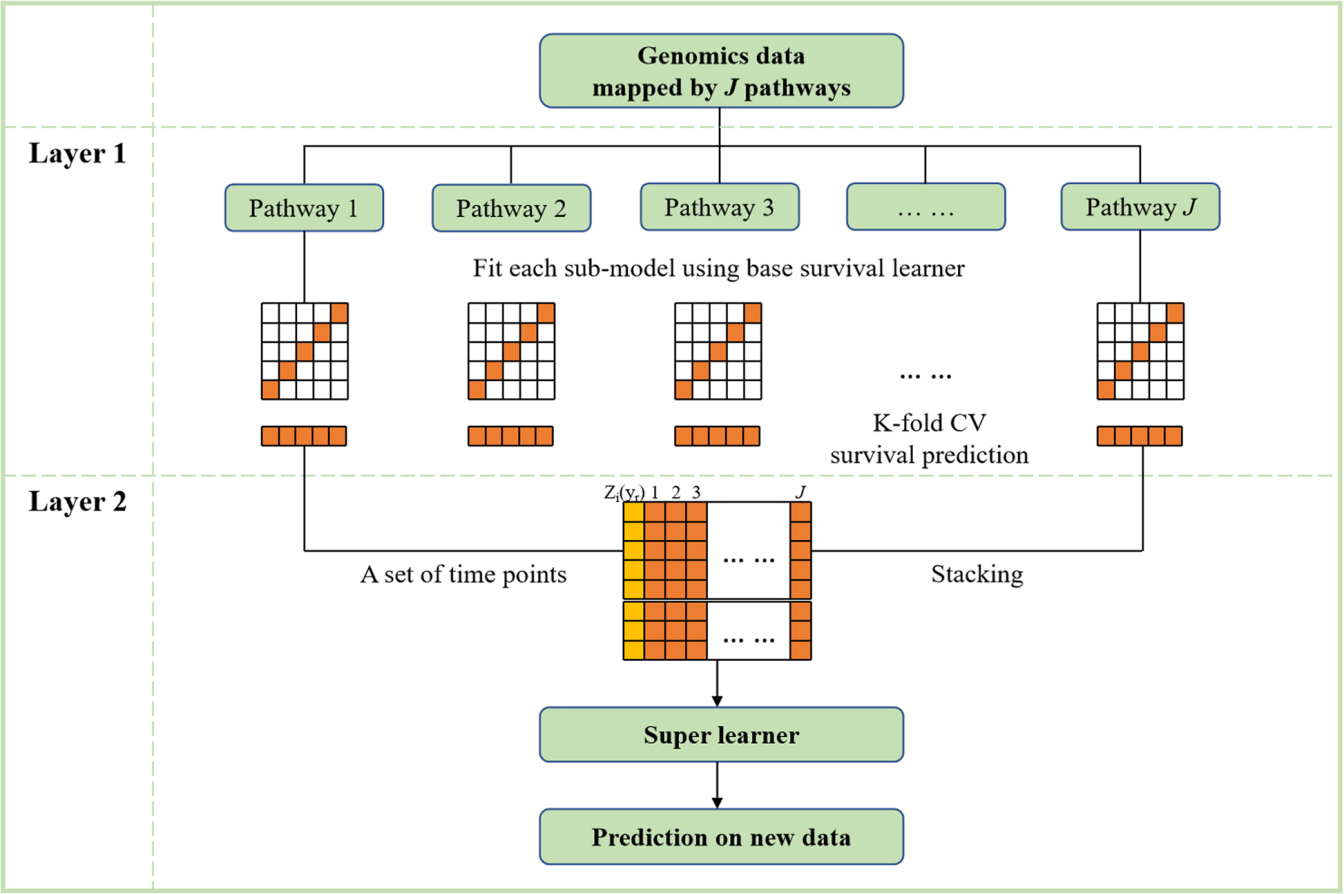
The algorithm flow plot of the proposed survival stacking model. CV: cross-validated

The proposed survival stacking method is a two-layer learning structure consisting of multiple base learners (sub-models) and a super learner (meta-model). See Fig. 1 for the framework flow.

We first transform the genomics data into *J* sub-data containing genes in each pathway. Then, in the first layer, sub-models are independently trained for each sub-data. The resulting sub-models represent the predictive capacity of pathways. To mitigate overfitting, we calculate the cross-validated survival predictions based on sub-models. Specifically, in each pathway, samples of original data are randomly partitions into *K* subsets (folds) of (rough) equal size. The *k*^th^ fold is used as the validation data, *V*(*k*), while the remaining folds are the training data, *T*(−*k*), *k* = 1, 2, …, *K*. In the training data, penalty Cox model can be used to fit sub-model and the baseline hazard *h*_0_^−*k*^(*y*^−*k*^) can be estimated by the breslow method. Then the linear predictor (*lp*^*k*^) in the validation data is estimated by the fitted sub-model. The estimated survival probabilities ${\hat{S}}^k\left({y}^k|\boldsymbol{x}\right)$ in *V*(*k*) can be calculated using *lp*^*k*^ and *h*_0_^−*k*^(*y*^−*k*^), that is

1 $${\hat{S}}^k\left({y}^k|\boldsymbol{x}\right)={e}^{-{H}^{-k}\left({y}^{-k}\right)}$$

where ${H}^{-k}\left({y}^{-k}\right)={H}_0^{-k}\left({y}^{-k}\right)\times {e}^{lp^k}$, ${H}_0^{-k}\left({y}^{-k}\right)$ is cumulative baseline hazard, i.e. the integral of *h*_0_^−*k*^(*y*^−*k*^). The process is repeated for all *K* folds, yielding the CV predictive survival probabilities of all cases. For *J* sub-models, we can obtain *J* predictions ${{\hat{S}}_j}^{CV}\left(y|\boldsymbol{x}\right)=\sum_{k=1}^K{{\hat{S}}_j}^k\left({y}^k|\boldsymbol{x}\right),\kern0.5em j=1,2,\dots, J$. The second layer uses a super learner to fit the CV survival predictions of *J* sub-models over a set of time points. The resulting coefficients are the estimated weights ${\hat{w}}_j$ for *J* sub-models. The predictive survival function $\hat{S}\left(y|\boldsymbol{x}\right)$ can be estimated by combining the predictions of *J* sub-models ${\hat{S}}_j\left(y|\boldsymbol{x}\right)$ (refit in the original data) using the weights ${\hat{w}}_j$.

### Method to estimate weights ${\hat{w}}_j$

#### Linear combination approach

Typically, the predictive survival function $\hat{S}\left(y|\boldsymbol{x}\right)$ is a linear combination of the predictions of *J* candidate sub-models given as,

2 $$\hat{S}\left(y|\boldsymbol{x}\right)=\sum_{j=1}^J{\hat{w}}_j{\hat{S}}_j\left(y|\boldsymbol{x}\right)$$

We optimize the weights $\hat{w}$ by minimizing the IBS loss. The other loss function, such as, AUC-based loss, should be a favorable alternative [22]. IBS measures the squared distance between the probabilities and observed events over a set of time points *y*_1_, …, *y*_*s*_ [23], which can be written as,

3 $$\textrm{IBS}=\sum_{r=1}^s\sum_{i\in R\left({y}_r\right)}{\left\{{Z}_i\left({y}_r\right)-\sum_{j=1}^J{\hat{w}}_j{{\hat{S}}_j}^{(CV)}\left({y}_r|{\boldsymbol{x}}_i\right)\right\}}^2$$

where *R*(*y*_*r*_) represents patients who are still at risk at the time *y*_*r*_, *Z*_*i*_(*y*_*r*_) = *I*(*y*_*i*_ > *y*_*r*_). We can estimate $\hat{w}$ by minimizing IBS. Generally, the estimated weights ${\hat{w}}_j$ are constrained to non-negative for lower variance and better prediction. This constraint can be achieved by employing a nonlinear optimization algorithm based on the augmented Lagrange method which can be implemented in R function *solnp* [24]. Concerning the selection of time sets *y*_1_, …, *y*_*s*_, we use nine evenly spaced quantiles of the observed event distribution as Andrew Wey advocated [19].

#### Bayesian combination approach

In addition to the IBS solutions, if we treat the survival predictions of the sub-models as covariates, and treat the time-dependent status *Z*_*i*_(*y*_*r*_) (0 for dead and 1 for alive at each time point *y*_*r*_) as a binary outcome, the predicted survival can be expressed as,

4 $$E\left[\hat{S}\left(y|\boldsymbol{x}\right)\right]={h}^{-1}\left[{w}_0+\sum_{j=1}^J{\hat{w}}_j{\hat{S}}_j\left(y|\boldsymbol{x}\right)\right]$$

which is a generalized linear model (GLM). *h* is a link function such as a sigmoid function, to ensure the expected predicted survival probability to be 0–1.

##### Non-negative lasso (nLasso)

The advance of formula (4) is that we can add the *l*1 penalty term into the above GLM and thereby expanding the usage of the survival stacking, such as handling numerous sub-models (in a high-dimensional scenario), which is impractical for solnp.

It is well known that the Lasso is equivalent to a Bayesian hierarchical model with DE prior on coefficients [25], with coefficients qualified as non-negative in this study,

5 $${w}_j\mid s\sim DE\left({w}_j|0,s\right)=\frac{1}{2s}\mathit{\exp}\left(-\frac{w_j}{s}\right),\kern0.5em {w}_j\ge 0$$

where the scale, *s*, controls the degree of shrinkage; a smaller scale induces stronger shrinkage, driving the estimates of *w*_*j*_ toward zero. The weights fitted with nLasso are given by,

6 $$\hat{\boldsymbol{w}}=\mathit{\arg}\underset{\boldsymbol{w},{w}_j\ge 0}{\max}\left\{\mathit{\log}\left(l\left(\boldsymbol{w}\right)\right)-\sum_{j=1}^J\frac{{\hat{w}}_j}{s}\right\}$$

The weights above can be estimated by the cyclic coordinate descent algorithm using the *glmnet* package in R. The restriction of *w* to be non-negative can be conveniently performed using the *glmnet* package.

##### Non-negative spike-and-slab lasso (nsslasso)

We further extended the non-negative DE prior to the non-negative spike-and-slab mixture DE prior (Supplementary Fig. 1),

7 $${w}_j\mid {s}_j\sim DE\left({w}_j|0,{s}_j\right)=\frac{1}{2{s}_j}\mathit{\exp}\left(-\frac{w_j}{s_j}\right),\kern0.5em {w}_j\ge 0$$

where *s*_*j*_ = (1 − *γ*_*j*_)*s*_0_ + *γ*_*j*_*s*_1_ is called the total scale parameter; *γ*_*j*_ is an indicator (*γ*_*j*_ ∈ {0, 1}) following a binomial distribution; *s*_0_ and *s*_1_ (*s*_1_ > *s*_0_ > 0) are the scale parameters for spike and slab distribution, respectively. *s*_1_ applies weaker compression to the pathways of strong effects and is usually fixed at a larger value, say *s*_1_ = 1; while *s*_0_ gives stronger compression to the pathways of weak effects (or even compress to zero) and is a flexible smaller value selected from a set of predefined candidate values via cross-validation. Usually, spike-and-slab Lasso is more adaptive than Lasso [26]. The weights can be estimated by the EM coordinate descent algorithm [26] using the *glmnet* package and the *BhGLM* package in R. The restriction of weights to be non-negative can also be performed with the *glmnet* package.

#### Artificial neural network

Considering that the ANN can act as a classifier and give restricted (non-negative) weights to the input data, we can use it as a super learner. ANN uses backpropagation algorithm and gradient descent algorithm to iteratively estimate the weights.

### Evaluation of model performance

In principle, the survival stacking model is a binary classification problem for a given time [21]. Here, we employed the time-dependent AUC and time-dependent Brier Score (BS), which calculate the AUC and BS of the objects in the risk set of any time point, as recomended by Robert Tibshirani [21]. The time-dependent AUC is used to examine a model’s ability to discriminate between different outcomes at a given time point. The time-dependent BS is used to measure the calibration performance at a given time point: $\textrm{BS}(y)=\frac{1}{n}\sum_{i=1}^n{\left({Z}_i(y)-\hat{S}\left(y|\boldsymbol{x}\right)\right)}^2$. We selected three evaluated time points, namely 25, 50, and 75% quantiles of the total observation time of the test data.

### Competitive statistical methods

In our proposed survival stacking model, Lasso Cox was used to build pathway-based sub-models. To combine sub-models, we used the solnp (implemented by R function *solnp*), nLasso/nsslasso (implemented in the package *glmnet* and *BhGLM*), and ANN (implemented using TensorFlow library (2.3.0) of Python (3.7), the weights can be limited to non-negative by using kernel_constraint = non_neg()) as super learners. The fitting process of ANN see Supplementary Fig. 2 & 3. For time points, we used nine evenly spaced quantiles of the observed event distribution, that is {0, 0.125, 0.25, 0.375, 0.5, 0.625, 0.75, 0.875, 1}. We compared the performance of our proposed method with several existing single-model approaches, including the widely used Lasso Cox regression (*glmnet*) [27] and extensions that incorporate the group structures: the group spike-and-slab Lasso (gsslasso) (*BhGLM)* [28], overlap group Lasso (grlasso), overlap group cMCP, and overlap group smoothly clipped absolute deviation (grSCAD) (*grpregOverlap*) [29]. The performance of these methods was evaluated using simulated and real-world data. All single-model methods are executed using default parameters. All analyses were performed using the R (4.1.3) software on *Dale T7920 INTEL Windows 10 Gold 5117 CPU @ 2.00GHz*.

## Simulation study

### Simulation design

The present study designed six scenarios with varied theoretical generalized *R*^*2*^ and covariate coefficients (*β*) (Table 1). In each scenario, we generated two homogeneous datasets with equal sample sizes, one for training data and the other for test data. To assess the performance of the methods, we conducted 100 duplicated runs in each scenario and calculated the average results for comparison. This process is conducted using the R package *BhGLM*.

**Table 1:** The preset parameter settings of the six different simulation scenarios (*N* = 500, M = 1000)

Scenarios	Non-zero coefficients								Correlation coefficient *r*	Residual variance *σ*	^a^Adjusted generalized *R*^2^
	*β*_5_	*β*_20_	*β*_40_	*β*_210_	*β*_220_	*β*_240_	*β*_975_	*β*_995_			
1	0.80	−0.70	1.00	−0.90	−0.80	0.90	−1.00	0.70	0.60	0.35	0.50
2	0.80	−0.70	1.00	−0.90	− 0.80	0.90	−1.00	0.70	0.60	2.47	0.25
3	0.80	−0.70	1.00	−0.90	− 0.80	0.90	−1.00	0.70	0.60	5.14	0.10
4	0.80	−0.30	1.40	−0.90	− 0.80	0.90	−1.50	0.20	0.60	1.35	0.50
5	0.80	−0.30	1.40	−0.90	− 0.80	0.90	−1.50	0.20	0.60	3.50	0.25
6	0.80	−0.30	1.40	−0.90	− 0.80	0.90	−1.50	0.20	0.60	6.80	0.10

^a^Generalized *R*^2^ was obtained by fitting all variables (M = 1000) with the Cox regression model using a large sample (*N* = 20,000) and the adjusted *σ*

Specifically, in each dataset, we generated 500 samples, each with a survival outcome of *d*_*i*_ = {(*y*_*i*_, *δ*_*i*_)} and 1000 continuous covariates ***x***_*i*_ = (*x*_*i*, 1_, *x*_*i*, 2_, .., *x*_*i*, 1000_), for *i* = 1, 2, …, 500. The vector ***x***_*i*_ was randomly sampled from the multivariate normal distribution i.e. ***x***_*i*_~*N*(**0**, Σ), where Σ ∈ *ℝ*^1000×1000^ is the variance-covariance matrix. These covariates were assigned to 20 distinct groups, allowing for overlap between the groups, which is a mimic of pathway overlapping (Supplementary Table 1). The correlation coefficient *r* of covariates within groups was 0.6, and covariates between groups were independent. The observed survival time *y*_*i*_ was generated from the Weibull distribution [30]: ${y}_i={\left(-\frac{\mathit{\log}(U)}{\lambda exp\left({z}_i\right)}\right)}^{1/\nu }$ and the censored ratio was set to 50%. *δ*_*i*_ = 1 indicates the occurrence of events and *δ*_*i*_ = 0 indicates censored. The variable *U* was uniformly distributed over an interval between 0 to 1; We set the scale parameter *λ* = 3; shape parameter *ν* = 3; and intermediate variable *z*_*i*_ followed a univariate normal distribution *z*_*i*_~*N*(*μ*_*i*_, *σ*^2^), where ${\mu}_i={\sum}_{l=1}^{1000}{x}_{il}{\beta}_l$. *σ*^2^ denotes the residual variance, which was determined by fixing three theoretical generalized R^2^: 0.50, 0.25, and 0.10. We set eight non-zero covariate coefficients of two types: the absolute values of one range between 0.7 to 1, and the other range from 0.2 to 1.5.

### Results of the simulation

#### *Predicti*on *performance*

Table 2 summarizes the average time-AUC and time-BS of each method at 50% quantiles of the total observation time in the test data under six simulation scenarios. The results of the other two time points are shown in Supplementary Table 2. According to the simulation, the methods considering group structures, e.g., grlasso, and grSCAD, did not exhibit apparent advantages over Lasso Cox. However, gsslasso Cox and cMCP were competitive across all scenarios.

**Table 2:** Comparison of different methods with time-AUC and time-BS (mean(SD)) at 50% quantiles of the observed event distribution over 100 replicates under six simulation scenarios

	Scenario 1	Scenario 2	Scenario 3	Scenario 4	Scenario 5	Scenario 6
Time-AUC of penalty and group penalty methods						
Lasso	0.891(0.018)	0.731(0.027)	0.604(0.041)	0.873(0.020)	0.727(0.031)	0.612(0.081)
gsslasso	0.914(0.015)	0.727(0.029)	0.606(0.039)	0.879(0.022)	0.726(0.030)	0.613(0.036)
grlasso	0.859(0.023)	0.706(0.030)	0.563(0.048)	0.844(0.022)	0.698(0.033)	0.563(0.136)
grSCAD	0.845(0.024)	0.709(0.029)	0.565(0.048)	0.837(0.027)	0.700(0.033)	0.560(0.132)
cMCP	0.912(0.016)	0.729(0.028)	0.595(0.049)	0.874(0.021)	0.723(0.030)	0.610(0.081)
Time-AUC of pathway-stacking methods^a^						
solnp(Lasso)	0.874(0.018)	0.748(0.026)	0.627(0.041)	0.877(0.020)	0.747(0.031)	0.636(0.033)
nLasso(Lasso)	0.878(0.018)	0.752(0.026)	0.629(0.042)	0.879(0.019)	0.752(0.031)	0.636(0.033)
nsslasso(Lasso)	0.878(0.018)	0.752(0.026)	0.629(0.041)	0.879(0.019)	0.751(0.031)	0.636(0.033)
ANN(Lasso)	0.878(0.018)	0.754(0.025)	0.634(0.038)	0.879(0.019)	0.754(0.030)	0.638(0.033)
Time-BS of penalty and group penalty methods						
Lasso	0.118(0.007)	0.183(0.007)	0.203(0.031)	0.129(0.010)	0.181(0.008)	0.203(0.059)
gsslasso	0.101(0.007)	0.181(0.008)	0.202(0.007)	0.123(0.011)	0.178(0.009)	0.201(0.008)
grlasso	0.135(0.007)	0.190(0.007)	0.213(0.106)	0.144(0.009)	0.190(0.007)	0.215(0.119)
grSCAD	0.153(0.007)	0.192(0.006)	0.212(0.109)	0.165(0.011)	0.191(0.007)	0.215(0.115)
cMCP	0.102(0.007)	0.182(0.008)	0.206(0.086)	0.126(0.011)	0.181(0.008)	0.204(0.059)
Time-BS of pathway-stacking methods^a^						
solnp(Lasso)	0.140(0.008)	0.193(0.008)	0.215(0.007)	0.146(0.010)	0.191(0.008)	0.215(0.007)
nLasso(Lasso)	0.132(0.010)	0.189(0.011)	0.215(0.009)	0.133(0.012)	0.185(0.011)	0.216(0.009)
nsslasso(Lasso)	0.132(0.010)	0.189(0.011)	0.215(0.009)	0.133(0.012)	0.185(0.011)	0.216(0.009)
ANN(Lasso)	0.141(0.010)	0.201(0.008)	0.226(0.001)	0.146(0.011)	0.200(0.010)	0.225(0.007)

^a^In parentheses is the basic learner algorithm and out parentheses is the meta learner algorithm

All the four survival stacking methods outperformed the single-model methods except for Scenario 1 and 4. However, there was no significant difference between the four stacking methods based on AUC. The calibration of solnp(Lasso) performed well across all scenarios at 25 and 50% time points while nLasso(Lasso) / nsslasso(Lasso) performed superior at 75% time point. ANN(Lasso) performed moderately at the three time points. Of note, although the AUCs of the three time points are very close, the BS increases with the time.

#### Distribution of estimated weights

**Fig. 2:**
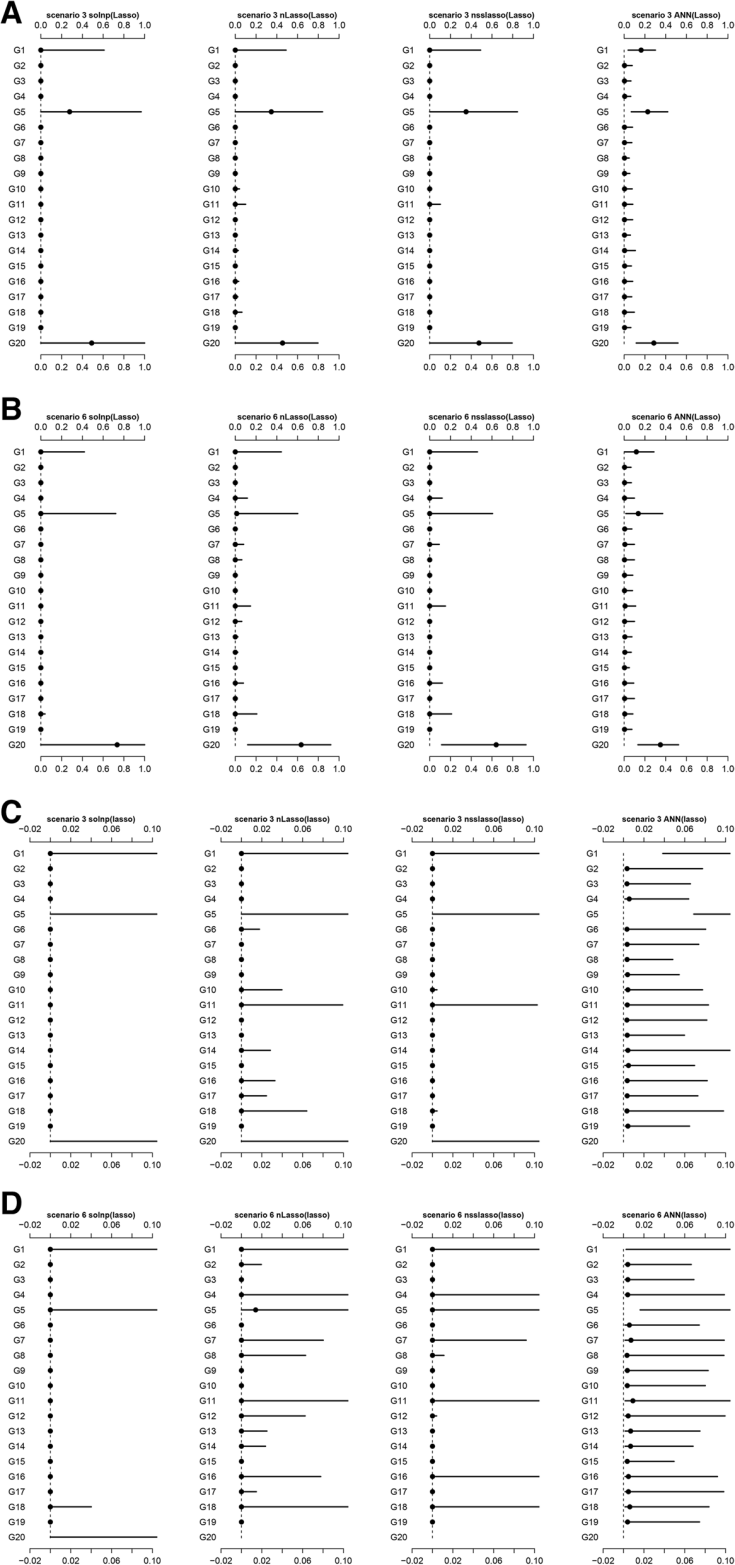
The distribution of weights estimated by stacking methods in different scenarios. (A) Scenario 3 with scale from 0 to 1; (B) Scenario 6 with scale from 0 to 1; (C) Scenario 3 with scale from 0 to 1; (D) Scenario 4 with scale from 0 to 1. The estimated weights are normalized. The black dot represents the median and the line represents the 5–95 quantile intervals

We further compared the estimated weights between super learners. Theoretically, the weights for group1, group5, and group20 should be non-zero due to the presence of relevant non-zero variables. In general, all of the four super learners consistently identified the non-zero weights across most scenarios (Fig. 2 and Supplementary Fig. 4). solnp(Lasso) did a good job of giving very small weights to zero weights (Fig. 2C**/D**) while ANN(Lasso) had the narrowest interval range of non-zero weights. nLasso(Lasso) and nsslasso(Lasso) presented moderate results.

## Applications to real data

We applied the proposed method to three real-world cancer datasets with survival records and large-scale gene expression profiles. For these datasets, gene expression data were standardized using *covariates* function in *BhGLM* package. We randomly partitioned the original data into two subsets of equal sample size: one for training models and the other for evaluating model performance. The process was repeated 100 times in case of casual results due to data split. To ensure a balanced response, we performed a log-rank test on the survival curves between training and test data and considered those with *P*_*log* − *rank*_ > 0.5 being balanced splits that would be retained for further analysis. Genes were mapped to pathways using genome annotation tools. More precisely, we first mapped gene symbols to Entrez Ids using *annotateI* package and then mapped genes to KEGG pathways (default parameter) using *clusterProfiler* package [31].

### TCGA breast cancer dataset

We obtained the transcriptome profiles (in TPM format) and the corresponding latest survival information for TCGA Breast Cancer (BRCA) from “GDC Data Portal” (https://portal.gdc.cancer.gov/). We selected the female samples that had both survival outcomes and gene expression profiles. Genes with > 50% of zero expression were filtered out and those with > 20% quantile variance were retained. Eventually, we ended up with a dataset consisting of 1060 samples and 13,745 genes. These genes were mapped to 140 pathways involving 3855 genes (see Supplementary Table 3).

Prior to the stacking process, we performed an initial pathways screening to identify those with potential predictive value. We fitted a Lasso Cox for all 140 pathways in the original data separately and obtained the C-index for each pathway. A total of 116 pathways had a C-index > 0.5. However, many of them were not predictive but introduced variance, which was detrimental to the ensembled prediction. We further constrained the enrolled candidate pathways to these with C-index > 0.55, resulting in 48 pathways for the subsequent analysis.

Table 3 summarizes the average time-AUC and time-BS at the three time points of various methods applied to BRCA dataset. In general, gsslasso and grlasso showed superior predictive performance over other single-model methods. Pathway-stacking methods outperformed single-model methods in terms of discrimination. The stacking methods also demonstrated a high calibration in the early and middle survival time. Among the survival stacking methods, solnp(Lasso) exhibited a preferable calibration consistently across time but inferior discrimination. Nsslasso(Lasso) had a favorable performance in the early and middle periods while ANN(Lasso) performed better discrimination at middle-late survival time.

**Table 3:** The measurements (mean(SD)) of penalty and group penalty methods and pathway-stacking methods for TCGA breast cancer dataset (*N* = 1060) by 100 times random spilt to training set (*N* = 530) and test set (N = 530)^a^

	Time at 25% quantiles of the observed event distribution		Time at 50% quantiles of the observed event distribution		Time at 75% quantiles of the observed event distribution	
Single-model methods	AUC	BS	AUC	BS	AUC	BS
Lasso	0.509(0.086)	0.064(0.088)	0.549(0.060)	0.093(0.074)	0.555(0.064)	0.151(0.048)
gsslasso	0.560(0.096)	0.030(0.039)	0.574(0.066)	0.064(0.034)	0.599(0.062)	0.133(0.023)
grlasso	0.569(0.082)	0.060(0.083)	0.582(0.064)	0.089(0.071)	0.595(0.063)	0.150(0.045)
grSCAD	0.543(0.068)	0.101(0.108)	0.544(0.058)	0.124(0.091)	0.561(0.060)	0.170(0.058)
cMCP	0.558(0.095)	0.123(0.113)	0.548(0.069)	0.143(0.095)	0.559(0.080)	0.183(0.060)
Pathway-stacking methods^b^						
solnp(Lasso)	0.600(0.069)	0.028(0.005)	0.605(0.060)	0.077(0.009)	0.608(0.050)	0.191(0.011)
nLasso(Lasso)	0.598(0.074)	0.028(0.005)	0.609(0.056)	0.078(0.009)	0.613(0.043)	0.190(0.013)
nsslasso(Lasso)	0.605(0.071)	0.028(0.005)	0.611(0.056)	0.078(0.009)	0.615(0.044)	0.190(0.013)
ANN(Lasso)	0.593(0.067)	0.028(0.005)	0.619(0.059)	0.077(0.009)	0.622(0.046)	0.204(0.011)

^a^We performed log-rank test of survival curves between training set and test set, and kept spilt sets by *p* > 0.5

^b^In parentheses is the basic learner algorithm and out parentheses is the meta learner algorithm

An advantage of nLasso and nsslasso is that they can identify important pathways owing to their sparsity nature. When applied to the whole dataset of TCGA BRCA, nsslasso(Lasso) and nLasso(Lasso) could select similar pathways. Nsslasso(Lasso) found three pathways including Huntington’s disease (*w* = 0.962), HIF-1 signaling pathway (relative weight, *w* = 0.076), and Leishmaniasis (*w* = 0.062) (see Supplementary Table 4). nLasso(Lasso) found four pathways including Huntington’s disease (*w* = 0.749), HIF-1 signaling pathway (*w* = 0.114), Leishmaniasis (*w* = 0.086), and Oxidative phosphorylation (*w* = 0.051), with the former three being selected by both methods.

### Metabric dataset

The Molecular Taxonomy of Breast Cancer International Consortium (METABRIC) data consists of comprehensive information on more than 2000 breast cancer patients, including clinical data, gene expression data, and mutation data. We obtained gene expression data and survival data from cBipPortal (https://www.cbioportal.org/). After data preprocessing (as described in 4.1), we obtained a dataset with 1420 samples and 19,494 genes. These genes were mapped to 146 pathways involving 3709 genes (see Supplementary Table 5).

After the pathways pre-screening, we included 138 of 146 pathways with a C-index > 0.60 for the following analysis. Among the single-model methods, grlasso still had the most superior predictive performance. Pathway-stacking methods showed favorable discrimination compared to grlasso (Table 4). nLasso(Lasso) and nsslasso(Lasso) performed well both in discrimination and calibration.

**Table 4:** The measurements (mean(SD)) of penalty and group penalty methods and pathway-stacking methods for METABRIC dataset (*N* = 1420) by 100 times random spilt to training set (*N* = 710) and test set (N = 710)^a^

	Time at 25% quantiles of the observed event distribution		Time at 50% quantiles of the observed event distribution		Time at 75% quantiles of the observed event distribution	
Single-model methods	AUC	BS	AUC	BS	AUC	BS
Lasso	0.705(0.022)	0.160(0.003)	0.679(0.018)	0.213(0.004)	0.651(0.020)	0.235(0.005)
gsslasso	0.701(0.022)	0.159(0.004)	0.675(0.017)	0.215(0.006)	0.653(0.019)	0.239(0.008)
grlasso	0.699(0.020)	0.160(0.003)	0.681(0.017)	0.213(0.004)	0.660(0.018)	0.235(0.005)
grSCAD	0.695(0.022)	0.162(0.004)	0.677(0.021)	0.215(0.005)	0.655(0.022)	0.235(0.006)
cMCP	0.697(0.024)	0.161(0.004)	0.671(0.020)	0.215(0.004)	0.644(0.021)	0.237(0.005)
Pathway-stacking methods^b^						
solnp(Lasso)	0.706(0.021)	0.162(0.003)	0.682(0.016)	0.222(0.003)	0.663(0.019)	0.235(0.005)
nLasso(Lasso)	0.712(0.020)	0.163(0.005)	0.688(0.016)	0.221(0.006)	0.668(0.019)	0.218(0.006)
nsslasso(Lasso)	0.712(0.020)	0.163(0.005)	0.688(0.016)	0.221(0.006)	0.669(0.019)	0.218(0.007)
ANN(Lasso)	0.718(0.020)	0.177(0.007)	0.692(0.016)	0.228(0.009)	0.671(0.019)	0.227(0.013)

^a^We performed log-rank test of survival curves between training set and test set, and kept spilt sets by p > 0.5

^b^In parentheses is the basic learner algorithm and out parentheses is the meta learner algorithm

The survival stacking model (nsslasso(Lasso)) fitted using the METABRIC dataset identified seven pathways (Supplementary Table 6). nLasso(Lasso) also found the same seven pathways: MAPK signaling pathway (W = 0.018), Focal adhesion (W = 0.041), Cellular senescence (W = 0.170), Choline metabolism in cancer (W = 0.125), Endocytosis (W = 0.014), Carbon metabolism (W = 0.311), Apoptosis (W = 0.215); and another two pathways: PPAR signaling pathway (W = 0.099) and p53 signaling pathway (W = 0.007).

### TCGA ovarian cancer dataset

Alike BRCA data, we acquired TCGA ovarian cancer (OV) dataset from the “GDC Data Portal”. After data preprocessing, we obtained a dataset with 415 samples and 13,764 genes. These genes were mapped to 124 pathways involving 3596 genes (see Supplementary Table 7).

After pre-screening, a total of 90 pathways had a C-index > 0.5 and the highest C-index was 0.58. We selected all 90 pathways for the following analysis. Table 5 showed that the pathway-stacking methods outperformed the single-model methods in prediction accuracy and variance (lower standard deviation especially for BS). The four stacking methods had similar and stable prediction performance.

**Table 5:** The measurements (mean(SD)) of penalty and group penalty methods and pathway-stacking methods for TCGA OV dataset (*N* = 415) by 100 times random spilt to training set (*N* = 207) and test set (*N* = 208)^a^

	Time at 25% quantiles of the observed event distribution		Time at 50% quantiles of the observed event distribution		Time at 75% quantiles of the observed event distribution	
Single-model methods	AUC	BS	AUC	BS	AUC	BS
Lasso	0.525(0.063)	0.154(0.041)	0.518(0.052)	0.230(0.040)	0.512(0.047)	0.241(0.137)
gsslasso	0.558(0.057)	0.112(0.012)	0.548(0.039)	0.223(0.014)	0.535(0.047)	0.241(0.030)
grlasso	0.547(0.054)	0.149(0.063)	0.551(0.050)	0.228(0.016)	0.548(0.052)	0.240(0.009)
grSCAD	0.549(0.057)	0.152(0.063)	0.556(0.049)	0.228(0.016)	0.548(0.048)	0.241(0.009)
cMCP	0.523(0.048)	0.171(0.068)	0.518(0.035)	0.235(0.015)	0.514(0.045)	0.244(0.010)
Pathway-stacking methods^b^						
solnp(Lasso)	0.562(0.058)	0.117(0.011)	0.559(0.042)	0.231(0.007)	0.547(0.041)	0.227(0.007)
nLasso(Lasso)	0.558(0.061)	0.117(0.011)	0.559(0.039)	0.236(0.010)	0.549(0.038)	0.232(0.008)
nsslasso(Lasso)	0.562(0.059)	0.117(0.011)	0.560(0.038)	0.236(0.010)	0.551(0.037)	0.232(0.009)
ANN(Lasso)	0.564(0.054)	0.117(0.011)	0.570(0.039)	0.239(0.005)	0.551(0.036)	0.227(0.006)

^a^We performed log-rank test of survival curves between training set and test set, and kept spilt sets by *p* > 0.5

^b^In parentheses is the basic learner algorithm and out parentheses is the meta learner algorithm

In application, nsslasso(Lasso) identified four pathways (Supplementary Table 8). nLasso(Lasso) found another two pathways, namely, Cell cycle (*w* = 0.038) and Proteasome (*w* = 0.079), in addition to the four pathways that were selected by nsslasso(Lasso) but with different weights: Influenza A (*w* = 0.360), Peroxisome (*w* = 0.268), B cell receptor signaling pathway (*w* = 0.128), and T cell receptor signaling pathway (*w* = 0.129).

## Discussion

The present study proposed a novel survival stacking strategy that can incorporate genome pathway information into the development of cancer prognosis models. This strategy demonstrated an advantage over existing methods that rely on a single group model (such as grlasso, grSCAD, gsslasso) by using a stacking method to improve prediction robustness. Additionally, we extended the super learner to hierarchical GLM and ANN, thereby enriching the combination of sub-models. Generally, solnp uses IBS as an optimization function to obtain a lower time-BS. Hierarchical Lasso and sslasso inherit the sparse property that makes them effective at handling multiple sub-models. The sslasso super learner could outperform Lasso in certain cases, while in others, the two methods performed similarly. The ANN method can capture more nonlinear relationships, leading to better prediction performance. However, it may also capture more noise information and overfit the data.

In the simulation study, stacking methods consistently exhibited superior performance in terms of discrimination over the methods using a single model, except for Scenarios 1 and 4. Scenarios 1 and 4 represented the situation of a higher theoretical generalized R^2^ or a small residual variance, in which the predictive information was easy to capture. The advantage of the stacking methods was not evident since these methods based on a single model had achieved a fairly well prediction. However, stacking methods demonstrated superior discrimination performance than any single model in the situation with more noise because they could borrow advantages from various models. Real-world data is typically characterized by a higher level of noise, which may account for the favorable performance of the proposed methods in the real-world data applications [32]. However, this may come at the expense of some calibration accuracy.

A noted point of the stacking using nsslasso is the interpretability of the resulting models. Firstly, the proposed stacking method demonstrates increased sensitivity in identifying disease-related pathways, which may be too subtle for gene-level models to detect [33]. Second, we implemented the methods considering group structure (e.g, gsslasso) to the real-world data (see Supplementary Table 9). The results indicated that while gsslasso exhibited good predictive performance, it did not effectively indicate pathway importance. Third, unlike Lasso which imposes an equal penalty on all coefficients, sslasso adaptively employed weak compression to strong effects and strong compression to weak effects [33]. We observed that sslasso tended to retain fewer pathways, while Lasso prefers to include more pathways with small effects. For instance, nsslasso(Lasso) identified several important pathways in METABRIC dataset, such as cellular senescence, choline metabolism in cancer, carbon metabolism, apoptosis, and PPAR signaling pathway. These pathways are deeply involved in the cell cycle and carcinogenesis process [34, 35]. nLasso(Lasso) could find two additional weak signal pathways, namely MAPK and p53 signaling pathways. These two popular pathways are associated with the prognosis of breast cancer [36, 37]. However, many MAPK family genes and TP53 are also contained in the other four pathways, indicating limited information that the two pathways can provide (Supplementary Table 6). Similarly, Huntington’s disease pathway identified in TCGA BRCA contains TP53. Huntington’s disease seems to be unrelated to the prognosis of breast cancer. However, several epidemiology studies have shown a lower risk of cancer among patients with Huntington’s [38–40]. Additional research has delved into their relationship at the molecular level, including the impact of Huntington and ErbB2/HER2 signaling on the development and metastasis of breast cancer [41, 42].

In total, the proposed methods possess advantageous features in identifying pathways that offer prognostic information. Also, the weights assigned to these sub-models (based on pathways) signify their predictive significance. We anticipate that focused research on these prioritized pathways will aid in discovering cancer targets. Another obvious property of the pathway-based stacking strategy is that sub-models are constructed independently, circumventing the gene-overlapping issue. In addition, one commonality of the stacking methods is having an improved discrimination than the single-based models, which may help identify high-risk patients. A limitation of our approach is that it takes more time due to the CV procedure in the sub-model construction. But the cost pays off in the more robust and accurate prediction. Last but not least, although the proposed survival stacking strategy is based on a two-level process of gene-pathway structure, our ideas can be naturally generalized to other biological processes with similarly hierarchical levels.

### Supplementary Information

**Supplementary Material 1.**

**Supplementary Material 2.**

## Data Availability

We acquired the dataset for Breast Invasive Carcinoma (ldentifier/Accession Number: TCGA-BRCA) from the TCGA (The Cancer Genome Atlas) database, accessible at https://portal.gdc.cancer.gov/projects/TCGA-BRCA. We obtained another breast cancer dataset with the identifier “Breast Invasive Ductal Carcinoma” from METABRIC (Molecular Taxonomy of Breast Cancer International Consortium, https://www.cbioportal.org/study/summary?id=brca_metabric). We acquired the dataset for Ovarian Cancer (ldentifier: TCGA-OA) from the TCGA database, accessible at https://portal.gdc.cancer.gov/projects/TCGA-OA. The main code for the proposed method is freely available on the GitHub website at: https://github.com/JasonLnzi/A-Bayesian-Stacking-Modeling-Method-for-Survival-Prediction-Using-High-dimensional-Data/tree/main.

## Abbreviations

Lasso: Least absolute shrinkage and selection operator
grlasso: Group Lasso
cMCP: Composite minimax concave penalty
CV: Cross-validated
IPCW-BS: Inverse probability-of-censoring-weighted Brier Score
IBS: Integrated Brier Score
BhGLM: Bayesian hierarchical generalized linear model
DE: Double-exponential
ANN: Artificial neural network
lp: Linear predictor
GLM: Generalized linear model
sslasso: Spike-and-slab Lasso
nsslasso: Non-negative spike-and-slab Lasso
AUC: Area under the ROC curve
BS: Brier Score
grSCAD: Overlap group SCAD
KEGG: Kyoto Encyclopedia of Genes and Genomes
TCGA: The Cancer Genome Atlas
BRCA: Breast cancer
METABRIC: Molecular Taxonomy of Breast Cancer International Consortiu
OV: Ovarian cancer
