# Supplementary material for: A novel non-negative Bayesian stacking modeling method for Cancer survival prediction using high-dimensional omics data
Source: BMC Med Res Methodol. 2024 May 3;24:105. doi: 10.1186/s12874-024-02232-3 (PMC11067084; doi:10.1186/s12874-024-02232-3)
Supplement: Supplementary file 1 — Supplementary Material 1. [file 12874_2024_2232_MOESM1_ESM.docx]

CONTENT

**Supplementary figures**

[Supplementary Figure 1.](#_Toc72662154) **[Diagram of non-negative spike-and-slab mixed prior distribution](#_Toc72662154)** [1](#_Toc72662154)

[Supplementary Figure 2.](#_Toc72662154) **[The fitting process of ANN in simulation study](#_Toc72662154)** [2](#_Toc72662154)

[Supplementary Figure 3.](#_Toc72662154) **[The fitting process of ANN in real world data](#_Toc72662154)** [3](#_Toc72662154)

[Supplementary Figure 4.](#_Toc72662154) **[The distribution of weights estimated by stacking methods in different scenarios](#_Toc72662154)** [4](#_Toc72662154)

**
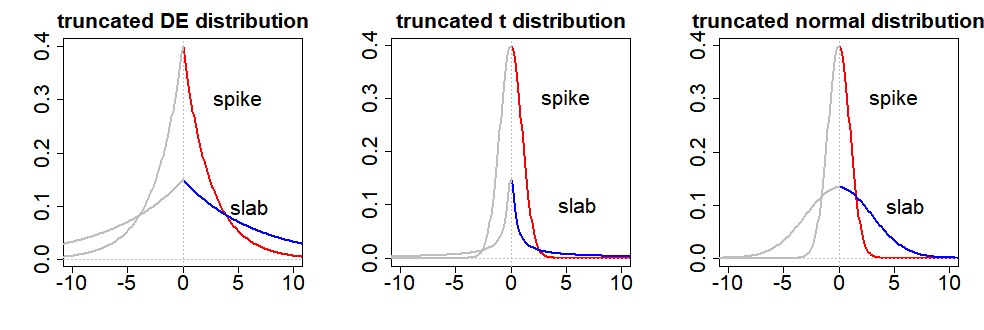
**

**Figure S1: Diagram of non-negative spike-and-slab mixed prior distribution.** The left panel is a truncated DE distribution with obvious peaks and gentle slop compared to truncated t distribution (the middle panel) and truncated normal distribution (the right panel).

**
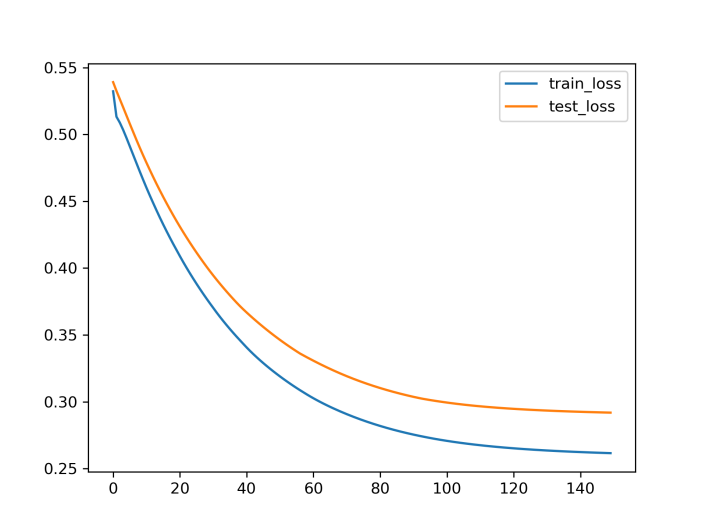

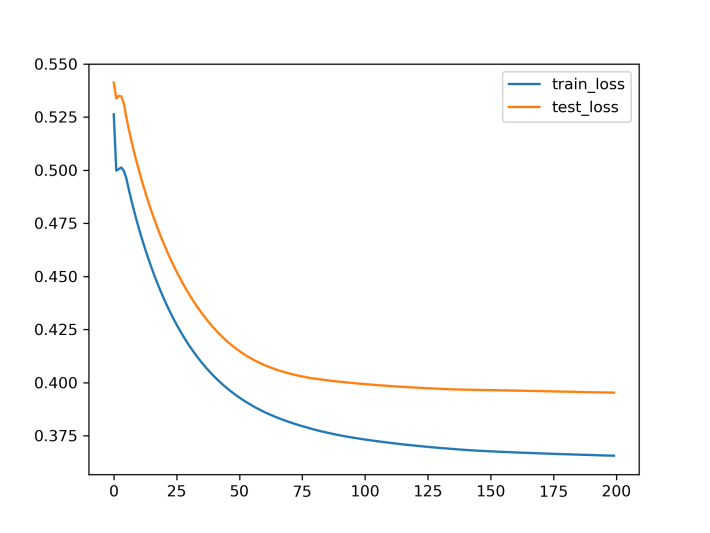

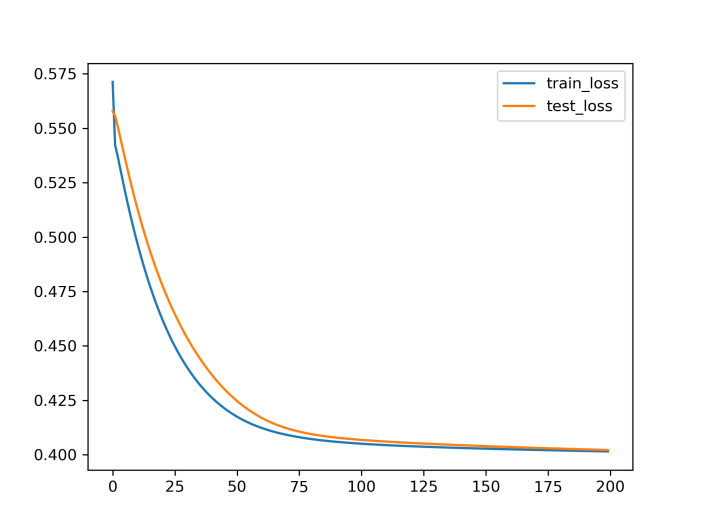
**

**A B C**

**Figure S2:** **The fitting process of ANN in simulation study.** (A): Scenario 1 & 4. One layer one node; activation = 'sigmoid'; optimizer = keras.optimizers.Adam(lr = 0.001); batch size = 32; epochs = 150. (B): Scenario 2 & 5. One layer one node; activation = 'sigmoid'; optimizer = keras.optimizers.Adam(lr = 0.002); batch size = 32; epochs = 200. (C): Scenario 3 & 6. One layer one node; activation = 'sigmoid'; optimizer = keras.optimizers.Adam(lr = 0.002); batch size = 32; epochs = 200.

**
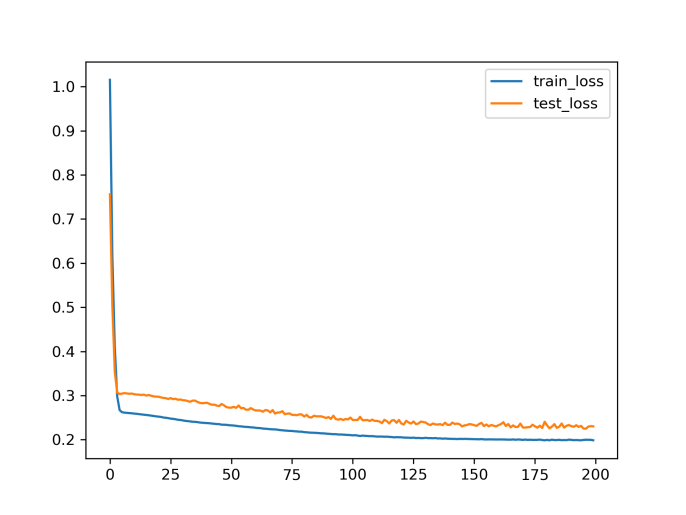

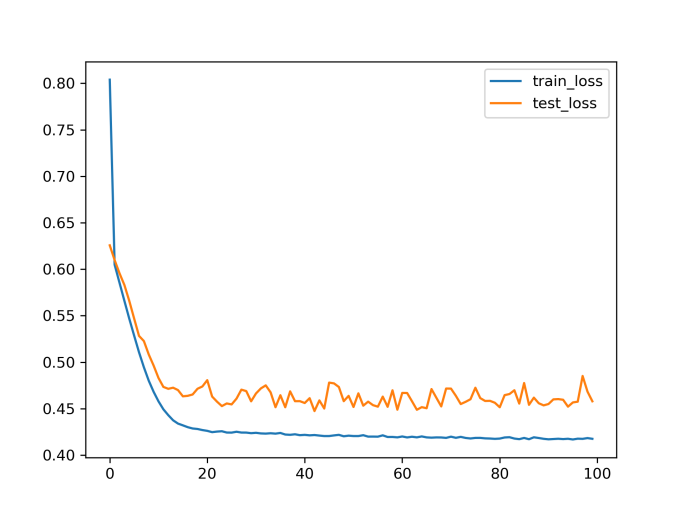

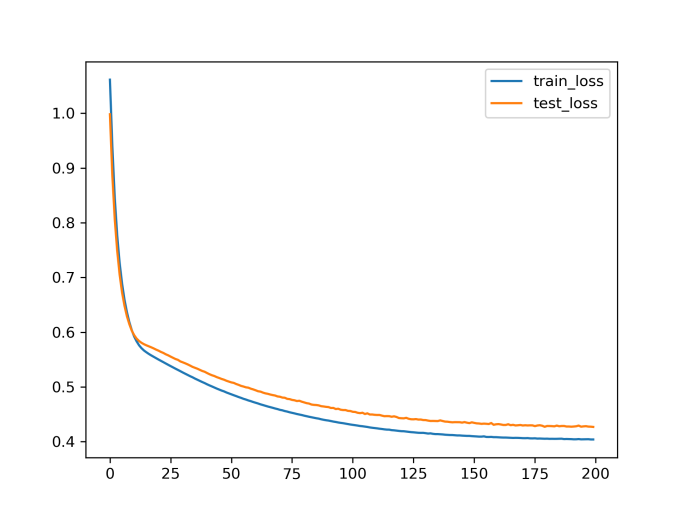
**

**A B C**

**Figure S3:** **The fitting process of ANN in real world data.** (A): TCGA BRCA. Three layer, 1 node, 32 nodes, 1 node; activation = 'relu', activation = 'relu', activation = 'sigmoid'; optimizer = keras.optimizers.Adam(lr = 0.0005); batch size = 32; epochs = 200. (B): METABRIC. Three layer, 1 node, 64 nodes, 1 node; activation = 'relu', activation = 'relu', activation = 'sigmoid'; optimizer = keras.optimizers.Adam(lr = 0.0005); batch size = 32; epochs = 100. (C): TCGA OV. Three layer, 1 node, 16 nodes, 1 node; activation = 'relu', activation = 'relu', activation = 'sigmoid'; optimizer = keras.optimizers.Adam(lr = 0.00015); batch size = 32; epochs = 200.

**
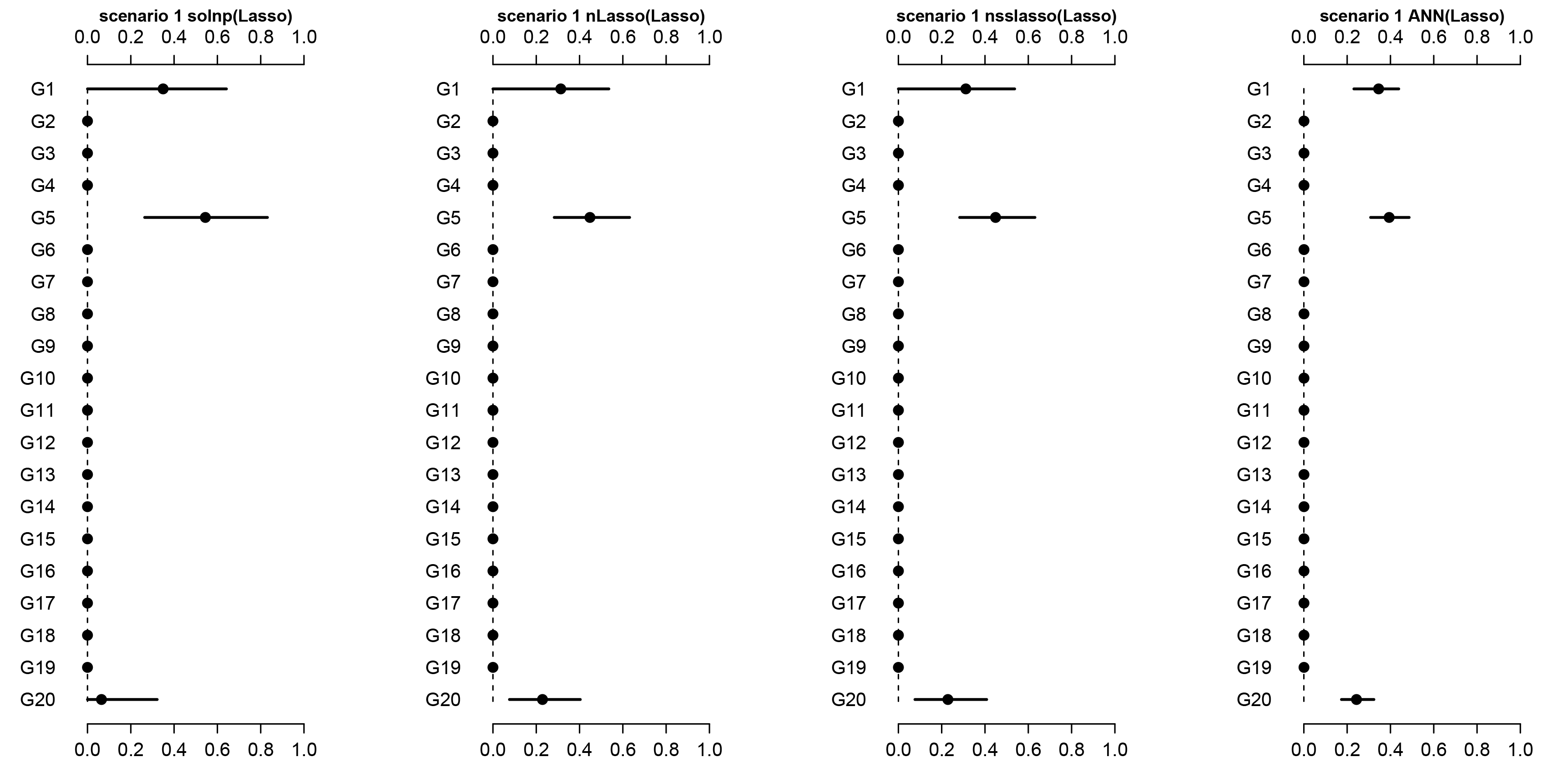
**

**A**

**
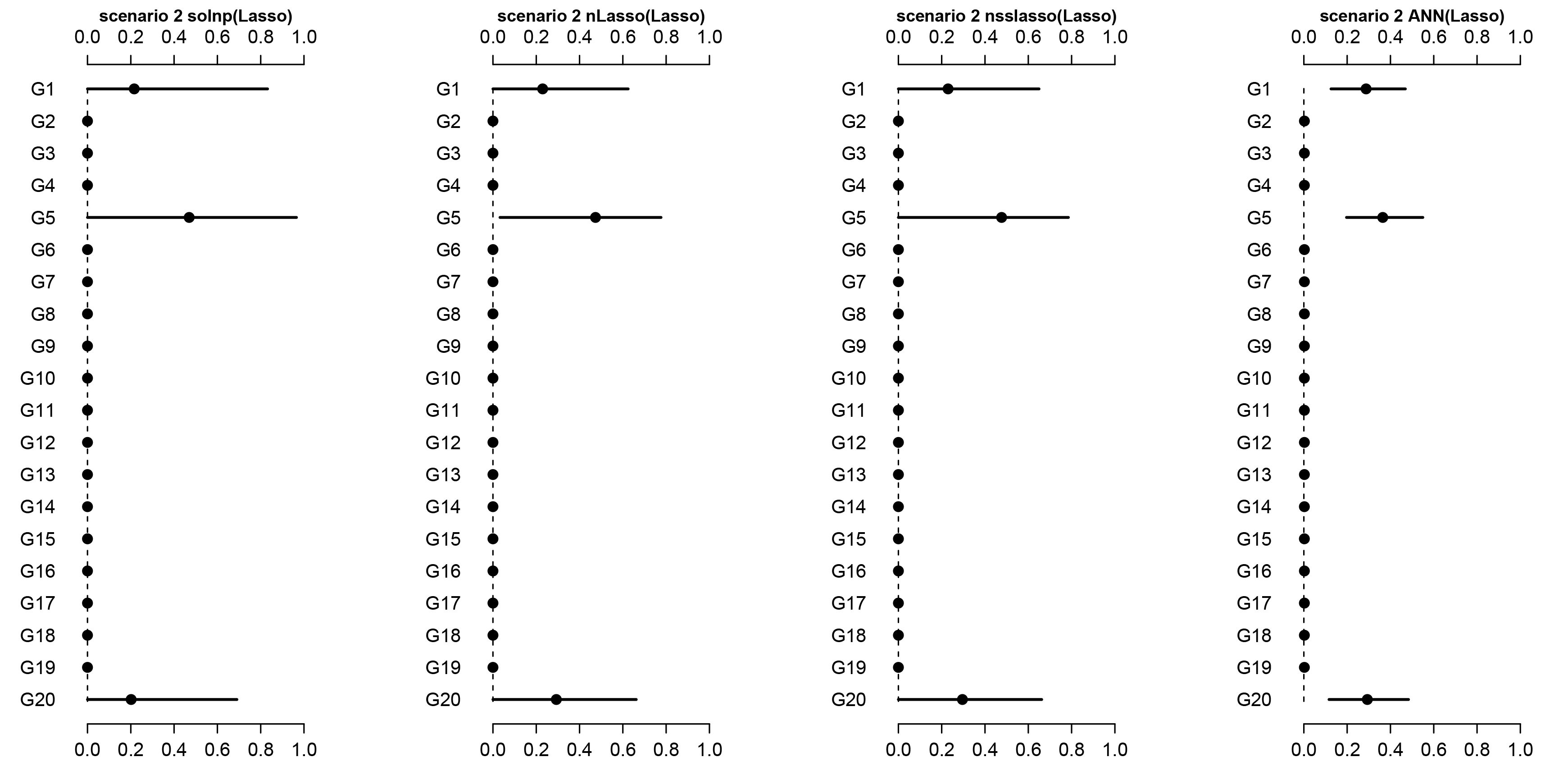
**

**B**

**
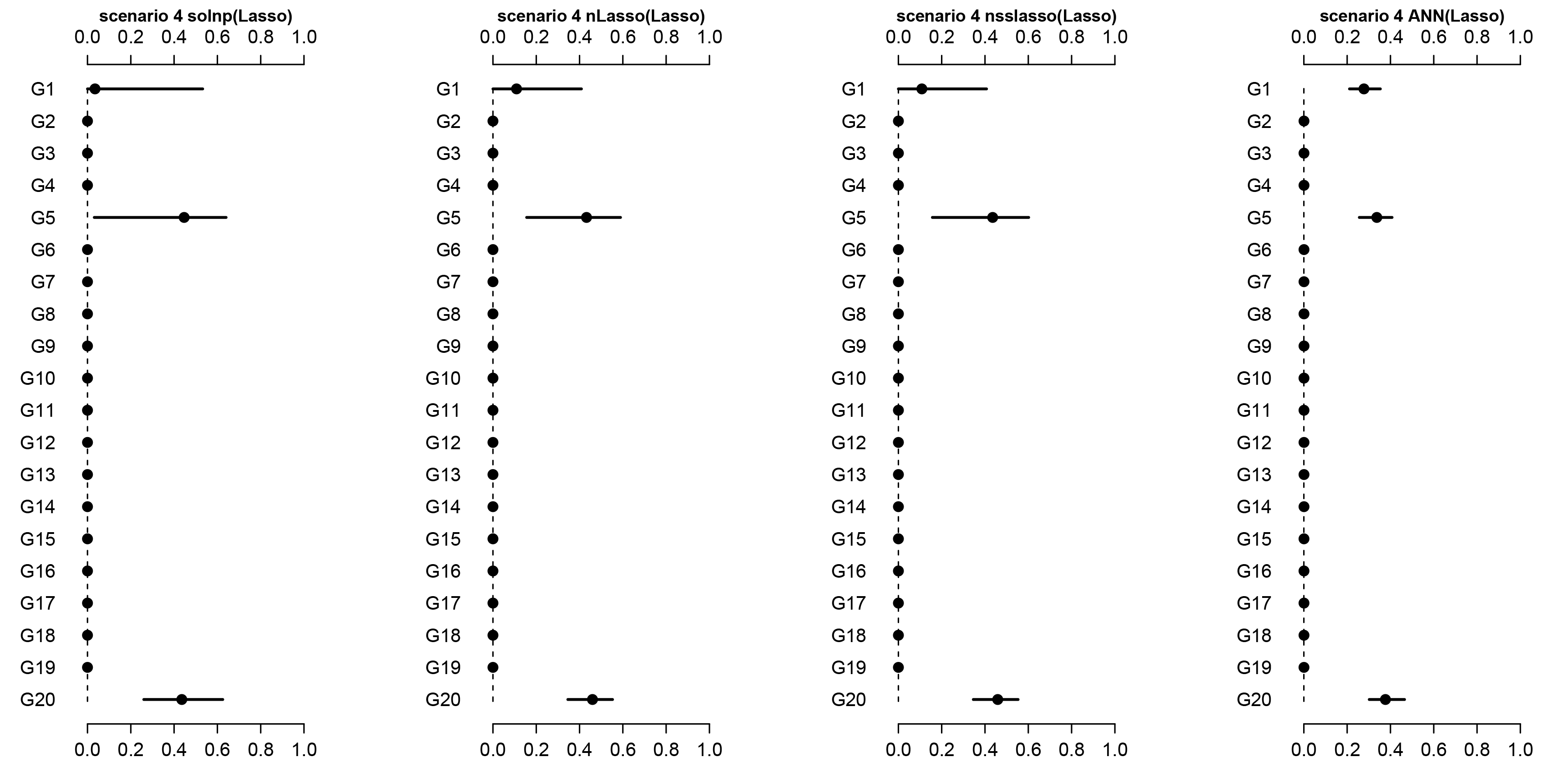
**

**C**

**
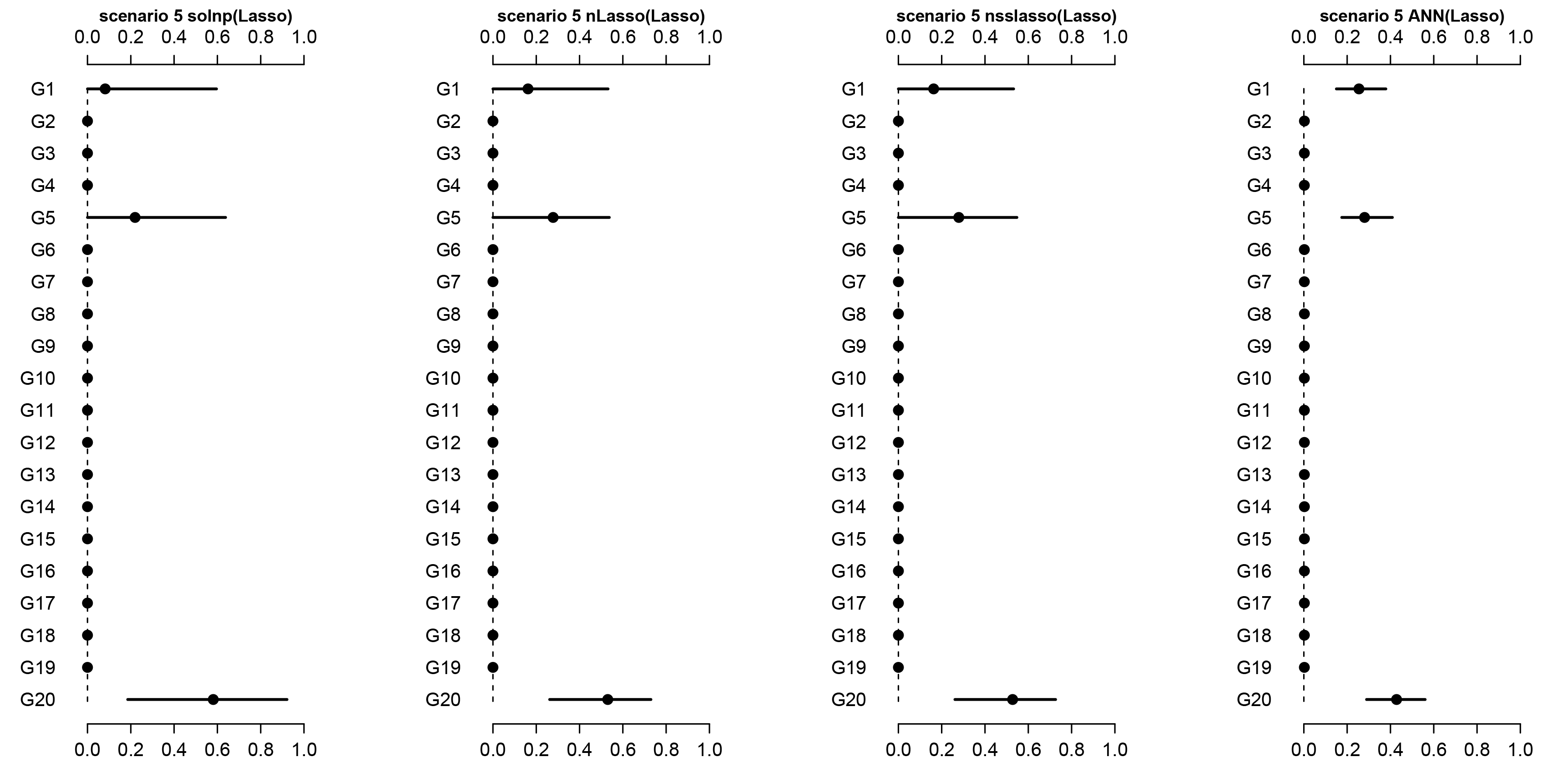
**

**D**

**Figure S4: The distribution of weights estimated by stacking methods in different scenarios.** (A) Scenario 1. (B) Scenario 2. (C) Scenario 4. (D) Scenario 5.

# Abbreviations used in the Supplementary Figures

DE: double-exponential
